# Beyond life extension– is it time for ‘Emergency Last Aid’ training?

**DOI:** 10.1186/s13049-025-01330-5

**Published:** 2025-02-04

**Authors:** Matthew Hooper, Marius Rehn

**Affiliations:** 1 MedSTAR Emergency Medical Retrieval Service, South Australian Ambulance Service, Adelaide, South Australia Australia; 2 Calvary Health Care, Adelaide, South Australia Australia; 3 School of Public Health and Tropical Medicine, James Cook University, Townsville, Australia; 4 Air Ambulance Department. Division of Prehospital Services, Oslo University Hospital, Oslo, Norway; 5 Institute of Clinical Medicine, University of Oslo, Oslo, Norway; 6 Department of Research and Development, Norwegian Air Ambulance Foundation, Oslo, Norway

As acute care clinicians operating in pre-hospital, emergency, and intensive care settings, our primary focus has always been on ensuring patient survival, reducing morbidity, and enhancing outcomes. Mortality and survival are key performance indicators that drive our practice. Yet, in our relentless pursuit of extending life, we are confronted daily with an undeniable reality: death. Whether sudden and traumatic, anticipated within the context of terminal illness or the culmination of overwhelming disease burden, we are deeply involved in the final moments of many patients who do not survive. Despite this inescapable truth, the management of dying patients in acute care environments remains remarkably underexplored in research and education, especially when compared to life-extending interventions.

At the recent London Trauma Conference, a compelling talk on managing death in pre-hospital and retrieval medicine sparked a robust discussion among international experts. The presentation highlighted critical gaps and opportunities in addressing this profoundly challenging aspect of care. Key questions emerged: What metrics can effectively evaluate the quality of pre-hospital care for patients who do not survive? How do our interactions with bystanders, families, and loved ones shape their experience of loss? Can we develop and standardise the skills needed to provide exceptional end-of-life care in acute settings? Most poignantly, how can we meet the challenge of navigating the most heart-wrenching scenarios, such as the witnessed traumatic death of a child or supporting someone aware of their own imminent death?

The feedback from senior pre-hospital and acute care clinicians worldwide was unanimous: there is a critical void in formal training and education for end-of-life care in pre-hospital and emergency settings. While the absence of death education has been acknowledged in pre-hospital care [1] and practical tools have been developed for emergency medicine [2], no internationally standardised, multi-specialty educational framework exists for doctors, paramedics, and nurses in these high-stakes environments.

“Last aid” training is not a novel concept [3], but its historical implementation has focused on improving death literacy in the general community and supporting non-medical caregivers. Just as first aid training laid the foundation for critical care medicine, there is now an urgent need for a formalised Emergency Last Aid (ELA) training program tailored to acute care clinicians. Such a program would fill a crucial gap, equipping professionals with the knowledge and skills to manage the complexities of end-of-life care in high-pressure scenarios.

An effective ELA course would encompass:

- **Recognising end-of-life care needs** in acute environments.
- **Breaking bad and catastrophic news.**
- **Understanding ethical considerations**, including autonomy and the momentum of potentially futile care.
- **Fostering leadership and human factors skills** for compassionate decision-making and bystander support.
- **Incorporating communication strategies** to facilitate meaningful interactions with families and teams.
- **Responding to anger** and other strong emotional reactions.
- **Developing strategies to enhance psychologically safe work practices.**
- **Honouring the impact of death** on patients, loved ones, and healthcare professionals.

The course would blend theoretical instruction with practical tools and strategies, including role-playing scenarios, supportive feedback, and peer learning. Experienced clinicians could share challenging cases, fostering group discussions and collaborative learning. This approach would build a shared understanding of how to navigate the emotional and clinical complexities of death and dying.

Our profession’s commitment to training and education has transformed outcomes for critically ill and injured patients. It is time to apply this same dedication to improving care for those who do not survive, as well as supporting their families, bystanders, and the clinicians involved. Every moment of end-of-life care carries immense emotional weight, and we have only one chance to get it right. The quality of our approach to death profoundly influences the experiences of everyone involved and leaves lasting emotional and psychological imprints.

In conclusion, pre-hospital and acute care settings urgently require a comprehensive educational framework to address the complexities of end-of-life care. Establishing an Emergency Last Aid training program would empower healthcare professionals to navigate these critical moments with skill, compassion, and confidence. By integrating this essential aspect of care into our education and practice, we can ensure that the quality of our care in death matches the excellence of our efforts to extend life. The time to act is now.

## Data Availability

No datasets were generated or analysed during the current study.
